# Chronic pain, fatigue, and emotional distress in female *FMR1* premutation carriers

**DOI:** 10.3389/fnmol.2026.1741854

**Published:** 2026-03-25

**Authors:** Dragana Protic, Maja Stojkovic, Randi Hagerman, Danijela Bascarevic, Jovana Ogrizovic, Sanja Dimitrijevic, Jovan Pesovic, Dusanka Savic-Pavicevic, Dejan Budimirovic

**Affiliations:** 1 Department of Pharmacology, Clinical Pharmacology and Toxicology, Faculty of Medicine, University of Belgrade, Belgrade, Serbia; 2 Special Hospital for Cerebral Palsy and Developmental Neurology, Belgrade, Serbia; 3 Department of Pediatrics, University of California Davis School of Medicine, Sacramento, CA, United States; 4 MIND Institute, University of California Davis Medical Center, Sacramento, CA, United States; 5 Institute for Rehabilitation, Belgrade, Serbia; 6 Center for Human Molecular Genetics, Faculty of Biology, University of Belgrade, Belgrade, Serbia; 7 Division of Child and Adolescent Psychiatry, Cincinnati Children’s Hospital Medical Center, Cincinnati, OH, United States

**Keywords:** anxiety, chronic fatigue, chronic pain, depression, fatigue assessment scale, *FMR1* gene premutation, ild care foundation (www.ildcare.nl), symptom impact questionnaire

## Abstract

**Introduction:**

Carriers of the *FMR1* gene premutation (PM) are at increased risk for Fragile X-associated PM Conditions (FXPAC). Some clinically significant symptoms can be further classified as Fragile X-associated Neuropsychiatric Disorders (FXAND). Many FXAND-related cases may go underrated and untreated. This study aimed to investigate the rates of FXAND-related symptoms among female PM carriers.

**Methods:**

The study was conducted at the Belgrade Fragile X Clinic on a clinical sample of 35 women with the PM and 35 controls using an adapted version of the Symptom Impact Questionnaire and the Fatigue Assessment Scale. The survey was designed to collect data on FXAND symptoms, including chronic pain, fatigue, anxiety, and depressive symptoms. Each symptom was self-rated by participants on a scale from 0 to 10. Data were analyzed using appropriate statistical methods.

**Results:**

Women with the PM (mean age: 44.51 ± 12.90 y.; 90.51 ± 22.04 CGG repeats) had statistically significant higher frequency and severity of chronic pain (*p* = 0.03; *p* = 0.02) and fatigue (*p* = 0.001 for both) in contrast to age-matched controls. Although the prevalence of anxiety symptoms was not significantly different between groups, the severity of anxiety symptoms were significantly higher in the PM group (*p* < 0.001), and was positively correlated with chronic fatigue (*p* = 0.003 vs. *p* = 0.27 in controls). Depressive symptom frequency and severity did not differ between groups (*p* = 0.47; *p* = 0.55), but there were a significant positive correlation between anxiety and depressive symptoms in the PM group (*p* = 0.003). Depressive symptoms were also positively correlated with chronic fatigue in the PM group (*p* = 0.02), but not in controls (*p* = 0.58). Compared to controls, PM carriers reported more frequently lower energy, poorer sleep, greater memory issues, cognitive difficulties, balance problems, and increased sensory sensitivity (*p* ≤ 0.001, all).

**Conclusion:**

Female PM carriers experience significantly higher frequency and severity of FXAND-related symptoms. Our findings of an association between fatigue, anxiety, and depressive symptoms highlight the need for comprehensive screening and underscore the importance of recognizing and treating individuals with FXAND.

## 1 Introduction

The *FMR1* (Fragile X Messenger Ribonucleoprotein 1) gene, located on the X chromosome at position Xq27.3, encodes the *FMR1* protein (FMRP), an RNA-binding protein essential for proper synaptic development and plasticity (Richter and Zhao, 2021). The *FMR1* gene contains between 5 and 44 CGG trinucleotide repeats in its 5′ untranslated region (UTR), with 29 and 30 repeats being the most common (Richter and Zhao, 2021). However, when CGG repeats undergo abnormal expansion, different classes of expanded alleles are recognized: (i) intermediate or gray zone (45–54 CGG repeats) with unclear clinical significance, (ii) premutation (PM) with 55–200 CGG repeats, and (iii) full mutation (FM) with ≥ 200 CGG repeats (Maddalena et al., 2001; Wheeler et al., 2014). The methylation of the FM and other epigenetic modifications leads to the silencing of the *FMR1* gene, resulting in the loss of FMRP production. Clinically, this condition is recognized as fragile X syndrome (FXS) (Maddalena et al., 2001; Wheeler et al., 2014). The absence of FMRP impairs synaptic plasticity, leading to abnormalities such as delayed dendritic spine maturation and altered synapse structure—hallmarks of FXS (Crawford et al., 2020). FXS is a rare genetic disorder, occurring in approximately 1 in 4,000 males and 1 in 8,000 females, and it is the most common cause of inherited intellectual disability (ID) and autism spectrum disorder (ASD) (Hunter et al., 2014). In contrast, the PM of the *FMR1* gene is relatively common in the general population, and its prevalence varies across studies (Seltzer et al., 2012a; Tassone et al., 2013; Hunter et al., 2014; Hnoonual et al., 2024). Combining data from previously published studies, the estimated prevalence of the PM in the general population ranges from 1 in 250 to 1 in 850 males and 1 in 110 to 1 in 300 females (Seltzer et al., 2012a; Tassone et al., 2013; Hunter et al., 2014; Hagerman et al., 2017; Hnoonual et al., 2024).

While PM carriers do not develop FXS, some of them experience symptoms or conditions related to the PM (Tassanakijpanich et al., 2021; Tassone et al., 2023). The condition’s complexity, ranging from no symptoms to neuropsychiatric or physical issues, makes it challenging to find terminology that accurately reflects everyone’s experience without causing confusion or exclusion. Terms like “mutation” or “disorder” can carry negative connotations or imply illness, which many in the community find stigmatizing or misleading, especially for those who do not experience significant symptoms (Tassone et al., 2023). On the other hand, a carrier can suggest that a person is unaffected by the PM, even when they may experience symptoms (Tassone et al., 2023). Clinicians and researchers prioritize scientific precision, while individuals and families seek terms that are accessible and non-discriminatory. Terminology also evolves, which can lead to inconsistency. Nowadays, the most used term for such a group of conditions is the “Fragile X PM-associated conditions” (FXPAC) as an umbrella term that encompasses the range of involvement from the PM (Johnson et al., 2020; Tassone et al., 2023).

FXPAC encompasses several clinical entities: FXTAS (Fragile X-associated Tremor/Ataxia Syndrome)—a neurodegenerative disorder characterized by tremors and ataxia in older men and some women; FXPOI (Fragile X-associated Primary Ovarian Insufficiency)—premature ovarian insufficiency in women; and FXAND (Fragile X-associated Neuropsychiatric Disorders)—a group of neuropsychiatric disorders that can occur in the PM carriers of all ages (Tassone et al., 2023). The pathophysiological mechanisms underlying FXPAC involve the toxic effects of elevated levels of *FMR1* mRNA, which positively correlate with the CGG repeats in the PM range, as well as mitochondrial dysfunction (Tassone et al., 2007; Giulivi et al., 2016).

FXAND represents a relatively under-recognized clinical category within the FXPAC spectrum, encompassing a wide range of symptoms, including anxiety, depression, attention disorders (e.g., ADHD), autistic traits, obsessive-compulsive symptoms, sleep disturbances, chronic pain, chronic fatigue, etc. (Hagerman et al., 2018; Aishworiya et al., 2022). These manifestations may be present in children, adolescents, and adults carrying the PM, regardless of gender (Hagerman et al., 2018; Aishworiya et al., 2022). In clinical practice, FXAND symptoms are often underdiagnosed or misattributed to other neuropsychiatric conditions, highlighting the need for a better understanding and their timely identification (Tassone et al., 2023).

Although awareness of FXAND has increased in recent years, systematic data on chronic pain, fatigue, and emotional distress in female PM carriers remain limited and under-integrated into clinical practice. By focusing on these frequently overlooked yet clinically impactful symptom domains, the present study aims to expand the current understanding of the FXAND phenotype in female PM carriers. Improved characterization of these features has the potential to inform earlier identification, refine screening strategies, and support more comprehensive counseling and clinical management approaches tailored to women with the PM. Specifically, this study aimed to investigate the presence, severity, and interrelationship of FXAND-related symptoms such as chronic pain, fatigue, anxiety, and depressive symptoms among female PM carriers.

## 2 Materials and methods

### 2.1 Participants

The prospective study enrolled adult females who were divided into two groups: (i) the PM group, which included 35 unrelated female PM carriers (CGG repeat size 55–200), and (ii) the control group, which included 35 age-matched females who had a number of CGG repeats in the *FMR1* gene within the normal range. The number of CGG repeats in the *FMR1* gene in all participants was determined via PCR approaches using AmplideX^®^ PCR/CE *FMR1* Kit (Asuragen, Austin, TX, United States) (Chen et al., 2010; Filipovic-Sadic et al., 2010). Females, both PM carriers and controls, were enrolled after genetic testing, which was conducted as part of cascade screening following genetic counseling for a child or sibling diagnosed with FXS. Specifically, control females were members of families with fragile X, but they tested negative for the *FMR1* mutation and did not have children with ID and/or ASD. Exclusion criteria included FXTAS.

Ethical approval for the study was obtained from the IRB at the Special Hospital for Cerebral Palsy and Developmental Neurology (SHCPDN) in Belgrade, Serbia, where the Fragile X Clinic is established (No. 221; 691).

### 2.2 Data collection

During this observational study, data were collected through structured, in-person interviews by a clinician-researcher with expertise in the field of fragile X, with participant responses recorded anonymously. The interviewer was aware of the participant group’s status during the assessment. The structured, in-person interviews were based on a questionnaire that was developed using two instruments: the Symptom Impact Questionnaire (SIQR), the version of the Revised Fibromyalgia Impact Questionnaire (FIQR), and the Fatigue Assessment Scale (FAS) (Michielsen et al., 2003; Bennett et al., 2009). These instruments were selected for their strong psychometric properties, quick completion time (under 2 min), and ease of scoring. For this study, SIQR was used with permission of Mapi Research Trust (license No. 120280).^1^ In addition, the use of the FAS was permitted by the “ild care foundation”.^2^ Finally, the SIQR and FAS were combined into a single questionnaire, supplemented with basic socio-demographic items and self-assessment of the symptoms’ presence (symptoms of chronic pain, chronic fatigue, anxiety, and depression), and administered to all study participants. The final form of the questionnaire was conducted in accordance with the Strengthening the Reporting of Observational Studies in Epidemiology (STROBE) guidelines (Cuschieri, 2019).

Specifically, the data on chronic pain and related symptoms were collected by SIQR, which is commonly used in non-fibromyalgia patients and which does not use the word “fibromyalgia” (Bennett et al., 2009). The 2-page SIQR in English is freely available through the scientific article published in 2009 by Bennett et al. (2009). The SIQR comprises three domains. The domain 1 (function) measures the level of difficulty participants have experienced in performing activities over the past 7 days. Nine items were included in the domain 1, evaluating participants’ ability to brush or comb their hair (which is associated with shoulder girdle pain), walk continuously for 20 min, prepare a homemade meal, vacuum/scrub/sweep floors, lift and carry groceries, climb one flight of stairs, change bed sheets, sit in a chair for 45 min, and go grocery shopping. Domain 2 (overall impact): includes two items that assess the overall impact of any medical problems over the past 7 days—specifically, the perceived impact of medical problems on weekly goal accomplishment and the sense of being overwhelmed by medical problems and pain. Domain 3 (symptoms): 10 items were used to measure the severity of common pain-related symptoms, including pain, fatigue, stiffness, sleep quality, depression, memory issues, anxiety, tenderness, balance problems, and sensory sensitivities. Responses were recorded on a 0–10 numeric rating scale, in line with the original SIQR format, where 0 indicates no difficulty or symptom severity and 10 represents the worst possible difficulty or severity. Individual domain scores and a total SIQR score were calculated for each participant (Bennett et al., 2009).

Alongside the SIQR, the items from the FAS were incorporated to evaluate the presence and type of fatigue specifically (Michielsen et al., 2003). The FAS consists of 10 statements measuring both physical and mental fatigue. Participants rated their agreement with statements such as “I am bothered by fatigue,” “Physically, I feel exhausted,” “I have problems thinking clearly,” and “Mentally, I feel exhausted,” using a five-point Likert scale ranging from 1 (“never”) to 5 (“always”). Two positively phrased items: “I have enough energy for everyday life” and “When I am doing something, I can concentrate quite well”, were reverse scored to ensure consistent directionality, so that higher scores uniformly indicate greater fatigue. Sub-scores for total fatigue, mental fatigue, and physical fatigue were calculated according to the standard FAS scoring guidelines (Michielsen et al., 2003).

### 2.3 Statistical analyses

Descriptive statistics, including medians and ranges (min–max), were calculated for individual items, domain scores, and total scores. Chi-square tests were applied for frequency comparisons, while the independent t-test and Mann–Whitney U test were used for comparing continuous variables, depending on the normality of distribution. Specifically, multiple symptom domains were analyzed using separate Mann–Whitney U tests. No formal correction for multiple comparisons was applied to avoid an increased risk of Type II error given the small sample size and the correlated nature of the outcomes (VanderWeele and Mathur, 2018). Therefore, the possibility of Type I error should be considered when interpreting the results. Spearman’s rank correlation was used to assess relationships between variables. All analyses were performed using IBM SPSS Statistics, with a significance level at *p* < 0.05.

## 3 Results

### 3.1 Participants

This prospective study included 35 female participants aged from 23 to 75 years (mean age: 44.51 ± 12.90 y.; median 42 y.) who are carriers of the PM in the *FMR1* gene (PM group) and 35 age-matched females in the same age range who are non-carriers (mean age: 44.54 ± 12.91 y.; median 42 y.) as a control group. Among the women in the PM group, 22 (62.86%) had a child diagnosed with FXS. As expected, the number of CGG repeats in the *FMR1* gene was statistically significantly higher (*p* < 0.001) in the PM group (90.51 ± 22.04; median 85; range: 59–160) than in the control group (29.26 ± 3.15; median 30; range 21–35). There was also a statistically significant difference in body mass index (BMI) between groups, and PM carriers had a higher mean BMI compared to the control group (27.49 ± 5.30 in PM group vs. 23.55 ± 3.55 in control group; *p* = 0.001).

Chronic pain and chronic fatigue were statistically significantly more frequently self-reported in the PM group compared with the control group (PM group: 51.4% vs. control group: 25.7%; *p* = 0.027 for chronic pain; and PM group: 51.4% vs. control group: 11.4%; *p* = 0.001 for chronic fatigue). PM carriers more frequently reported anxiety symptoms than controls, but without statistical significance (PM group: 80.0% vs. control group: 62.9% *p* = 0.190). There was no statistically significant difference in the self-reported frequency of depressive symptoms between groups (PM group: 51.4% vs. control group: 62.9%; *p* = 0.469).

Demographic characteristics and self-reported symptom frequencies are presented in Table 1.

**Table 1:** Demographic characteristics and self-reported symptom frequencies of the study participants.

Participant characteristics	PM group	Control group	*p*
	*N* = 35	*N* = 35	
Age (mean ± SD)	44.51 ± 12.90	44.54 ± 12.91	0.993
CGG repeats (mean ± SD)	90.51 ± 22.04	29.26 ± 3.15	**<0.001**
CGG median (range)	85 (59–160)	30; (21–35)	**<0.001**
Having a child with FXS, N (%)	22 (62.86)	0 (0)	**/**
BMI (mean ± SD)	27.49 ± 5.30	23.55 ± 3.55	**0.001**
Chronic pain, N (%)	18 (51.4)	9 (25.7)	**0.027**
Chronic fatigue, N (%)	18 (51.4)	4 (11.4)	**0.001**
Anxiety symptoms, N (%)	28 (80.0)	22 (62.9)	0.185
Depressive symptoms, N (%)	18 (51.4)	22 (62.9)	0.469

PM, premutation in the *FMR1* gene, FXS, fragile X syndrome; N, number; BMI, body mass index. Bold: statistically significant *p* ≤ 0.05.

### 3.2 Manifestations of chronic pain and its impact on PM carriers’ functioning

In the present study, PM carriers reported statistically significantly higher pain levels than controls (median: 3, range: 0–10 in PM group vs. median: 1, range: 0–5 in control group; *p* = 0.021). Spearman correlation analysis revealed a moderate positive correlation between pain level and age in the PM group (*ρ* = 0.407, *p* = 0.002), while no such correlation was found in the control group (*ρ* = 0.040, *p* = 0.819). BMI was not significantly correlated with pain level in either the PM group (*ρ* = 0.188, *p* = 0.280) or the control group (*ρ* = 0.118, *p* = 0.500). In the PM group, pain level was not significantly correlated with CGG repeat number (*ρ* = −0.216, *p* = 0.213), nor was it significantly associated with having a child with FXS (*p* = 0.63). Pain levels did not significantly differ between PM carriers who have a child with FXS (median: 3, range: 0–10) and those without (median: 3.5, range: 0–10; *p* = 0.78).

In addition, there were no statistically significant differences between groups in terms of the reported impact of pain on weekly goal accomplishment or feelings of being overwhelmed by symptoms (median impact level: 0 in both groups; range: 0–10 in PM group and 0–3 in control group; *p* = 0.34 for both comparisons). A statistically significant difference in levels of pain-related limitations between groups was found for brushing or combing hair (PM group: median 0, range 0–10 vs. control: median 0, range 0–0; *p* = 0.006), whereas other activities did not show significant group differences. Detailed comparisons are presented in Table 2.

**Table 2:** Summary of symptom impact questionnaire (SIQR) results of the study participants.

Symptom Impact Questionnaire (SIQR) items	PM group	Control group	*p*
	*N* = 35	*N* = 35	
	median (min-max)	median (min-max)	
Domain 1			
1. Difficulty brushing or combing hair	0 (0–10)	0 (0–0)	**0.006**
2. Difficulty walking continuously for 20 min	0 (0–10)	0 (0–4)	0.106
3. Difficulty preparing a homemade meal	0 (0–10)	0 (0–2)	0.547
4. Difficulty vacuuming, scrubbing, sweeping floors	0 (0–10)	0 (0–4)	0.24
5. Difficulty lifting and carrying a bag full of groceries	1 (0–10)	0 (0–3)	0.059
6. Difficulty climbing one flight of stairs	0 (0–10)	0 (0–4)	0.097
7. Difficulty changing bed sheets	0 (0–10)	0 (0–3)	0.751
8. Difficulty sitting in a chair for 45 min	0 (0–10)	0 (0–3)	0.52
9. Difficulty going shopping for groceries	0 (0–10)	0 (0–5)	0.979
Domain 2			
1. Being able to accomplish most set goals for week	0 (0–10)	0 (0–3)	0.34
2. Overwhelmed by medical problems	0 (0–10)	0 (0–4)	0.328
Domain 3			
1. Pain level/severity	3 (0–10)	1 (0–5)	**0.021**
2. Energy level	5 (0–10)	3 (0–5)	**<0.001**
3. Level of stiffness	1 (0–7)	0 (0–4)	0.1
4. Quality of sleep	5 (0–10)	2 (0–5)	**0.001**
5. Level of depression	1 (0–9)	1 (0–6)	0.552
6. Level of memory problems	3 (0–10)	0 (0–3)	**<0.001**
7. Level of anxiety	4 (0–9)	1 (0–4)	**<0.001**
8. Level of tenderness to touch	3 (0–9)	0 (0–5)	0.092
9. Level of balance problems	2 (0–8)	0 (0–3)	**<0.001**
10. Level of sensitivity to loud noises, bright lights, odors, and cold	3 (0–10)	1 (0–5)	**0.001**

PM, premutation in the *FMR1* gene; N, number. Bold: statistically significant *p*-value ≤ 0.05.

### 3.3 Manifestations of chronic fatigue and its impacts on PM carriers’ functioning

The overall fatigue levels were statistically significantly higher in the PM group (median: 23, range: 11–49) compared to the control group (median: 17, range: 10–24; *p* = 0.001). Correlation analyses showed no significant relationship between fatigue level and age in the PM and control groups (PM group: *ρ* = 0.065, *p* = 0.713; control group *ρ* = −0.334, *p* = 0.060). BMI was not significantly correlated with fatigue in either group (PM group: *ρ* = 0.099, *p* = 0.571; control: *ρ* = −0.238, *p* = 0.168). In the PM group, fatigue level was not significantly correlated with CGG repeat number (*ρ* = −0.057, *p* = 0.713), nor was it associated with having a child with FXS (*p* = 0.83). Fatigue scores did not significantly differ between PM participants with (median: 26, range: 13–49) and without (median: 21.5, range: 11–36) a child with FXS (*p* = 0.41).

Specifically, the PM carriers reported being statistically significantly more affected by fatigue (*p* = 0.002), becoming tired more easily (*p* = 0.006), and feeling physically exhausted more frequently (*p* = 0.001) (PM group: median score 2, range 1–5 vs. control group: median score 2, range 1–3, for all parameters). The PM group also scored statistically significantly higher on both the mental fatigue (median: 9, range: 5–25 in PM group vs. 8, range: 5–12 in control group; *p* = 0.034) and physical fatigue subscales (median: 11, range: 6–24 in PM group vs. 9, range: 5–14 in control group; *p* < 0.001). Furthermore, PM carriers reported having sufficient energy for daily life less frequently than controls (*p* = 0.005). Detailed comparisons are presented in Table 3.

**Table 3:** Summary of fatigue assessment scale results of the study participants.

Fatigue Assessment Scale (FAS) items	PM group	Control group	*p*
	*N* = 35	*N* = 35	
	median (min-max)	median (min-max)	
1. Being bothered by fatigue	2 (1–5)	2 (1–3)	**0.002**
2. Getting tired very quickly	2 (1–5)	2 (1–3)	**0.006**
3. Doing little during the day	2 (1–5)	2 (1–3)	0.726
4. Having enough energy for everyday life	2 (1–5)	2 (1–3)	**0.005**
5. Feeling physically exhausted	2 (1–5)	2 (1–3)	**0.001**
6. Having problems starting things	2 (1–5)	2 (1–3)	0.081
7. Having problems thinking clearly	2 (1–5)	1 (1–2)	**0.001**
8. Feeling no desire to do anything	2 (1–5)	2 (1–3)	0.349
9. Feeling mentally exhausted	2 (1–5)	2 (1–3)	0.057
10. Being able to concentrate quite well when doing something	2 (1–4)	2 (1–2)	0.068
Score fatigue	23 (11–49)	17 (10–24)	**0.001**
Score mental fatigue	9 (5–25)	8 (5–12)	**0.034**
Score physical fatigue	11 (6–24)	9 (5–14)	**<0.001**

PM, premutation in the FMR1 gene; N, number. Bold: statistically significant *p* ≤ 0.05.

Finally, fatigue and pain levels were statistically significantly positively correlated in the control group (*ρ* = 0.557, *p* = 0.001), but not in the PM group (*ρ* = 0.321, *p* = 0.060).

### 3.4 Manifestations of anxiety symptoms and their impacts on PM carriers’ functioning

The severity of anxiety symptoms was statistically significantly higher in the PM group (median: 4, range: 0–9) compared to controls (median: 1, range: 0–4; *p* < 0.001) (Table 2). The severity of anxiety symptoms was not significantly correlated with age in the PM group (*ρ* = −0.257, *p* = 0.163). Interestingly a statistically significant moderate negative correlation was found in the control group (*ρ* = −0.631, *p* < 0.001). BMI was not significantly associated with anxiety symptoms in either group (PM group: *ρ* = −0.128, *p* = 0.462; control group: *ρ* = −0.213, *p* = 0.219). In the PM group, the severity of anxiety symptoms was not significantly correlated with CGG repeat number (*ρ* = 0.106, *p* = 0.543), nor was the presence of anxiety symptoms significantly associated with having a child with FXS (*p* = 0.38). The level of anxiety symptoms did not differ significantly between PM participants with a child with FXS (median: 4, range: 0–8) and those without (median: 4.5, range: 0–9; *p* = 0.72).

The presence of anxiety symptoms was not significantly associated with chronic pain in either the PM (*p* = 0.69) or control group (*p* = 0.78), and there was no significant correlation between the severity of anxiety symptoms and pain levels in either group (PM: *ρ* = 0.191, *p* = 0.273; control: *ρ* = 0.088, *p* = 0.613). In contrast, the presence of anxiety symptoms was statistically significantly associated with chronic fatigue in the PM group (*p* = 0.003) but not in the control group (*p* = 0.27). The severity of anxiety symptoms was positively correlated with fatigue levels in both groups (PM: *ρ* = 0.689, *p* < 0.001; control: *ρ* = 0.508, *p* = 0.002).

### 3.5 Manifestations of depressive symptoms and their impacts on PM carriers’ functioning

There was no statistically significant difference in the severity of depressive symptoms (median: 1, range: 0–9 vs. median: 1, range: 0–6; *p* = 0.55) (Table 2).

The severity of depressive symptoms was not significantly correlated with age in either group (PM: *ρ* = −0.162, *p* = 0.351; control: *ρ* = −0.188, *p* = 0.279). BMI was also not significantly associated with the severity of depressive symptoms (PM group: *ρ* = −0.044, *p* = 0.804; control group: *ρ* = 0.209, *p* = 0.869). In the PM group, the severity of depressive symptoms was not significantly correlated with CGG repeat number (*ρ* = −0.082, *p* = 0.639), nor was it significantly associated with having a child with FXS (*p* = 0.36). The severity of depressive symptoms did not differ significantly between PM carriers with a child with FXS (median: 2, range: 0–9) and those without (median: 0, range: 0–9; *p* = 0.45).

To assess symptoms commonly associated with depression, such as reduced motivation and cognitive difficulties, participants were asked about their motivation, ability to concentrate, and sense of accomplishment in daily life. Individuals with PM often reported feeling no desire to do anything, feeling mentally exhausted, feeling like they accomplish little in a day, and having more difficulty starting an activity more frequently than the control group, but none of these differences were statistically significant (PM group: median 2, range 1–5 vs. control group: median 2, range 1–3; all comparisons and *p* = 0.349; *p* = 0.057; *p* = 0.726; *p* = 0.081 respectively) (Table 3). Additionally, the PM group reported being able to concentrate well when engaged in an activity slightly less frequently than the control group, though this difference was also not statistically significant (median 2, range 1–4 vs. median 2, range 1–2; *p* = 0.068) (Table 3). There was a significant positive correlation between anxiety and depressive symptoms in both groups (PM: *p* = 0.003; control: *p* < 0.001), and the severity of depressive symptoms was statistically significantly positively correlated with anxiety levels in both PM (*ρ* = 0.697, *p* < 0.001) and control (*ρ* = 0.452, *p* = 0.006) groups.

The presence of depressive symptoms was not significantly associated with chronic pain in either group (PM: *p* = 0.74; control: *p* = 0.78). Depressive symptoms were statistically significantly associated with chronic fatigue in the PM group (*p* = 0.018), but not in the control group (*p* = 0.58). The severity of depressive symptoms was statistically significantly positively correlated with fatigue levels in both groups (PM: *ρ* = 0.505, *p* = 0.002; control: *ρ* = 0.697, *p* < 0.001).

### 3.6 Other FXAND-associated manifestations

As presented in Table 2, PM group also reported lower energy levels (PM group: median score 5, range 0–10 vs. control group: median score 3, range 0–5; *p* < 0.001), poorer sleep quality (PM group: median score 5, range 0–10 vs. control group: median score 2, range 0–5; *p* = 0.001), more pronounced balance issues (PM group: median score 2, range 0–8 vs. control group: median score 0, range 0–3; *p* < 0.001), and greater sensory sensitivity to loud sounds, smells, and cold (PM group: median score 3, range 0–10 vs. control group: median score 1, range 0–5; *p* = 0.001) (Table 2). On the other hand, there was no statistically significant difference between the two groups in terms of stiffness severity (PM group: median score 1, range 0–7 vs. control group: median score 0, range 0–4; *p* = 0.10), or tenderness to touch (PM group: median score 3, range 0–9 vs. control group: median score 0, range 0–5; *p* = 0.09) (Table 2).

Additionally, the PM group exhibited significantly greater memory difficulties (PM group: median score 3, range 0–10 vs. control group: median score 0, range 0–3; *p* < 0.001), significantly more frequent difficulty thinking clearly (PM group: median score 2, range 1–5 vs. control group: median score 1, range 1–2; *p* = 0.001) (Table 3) and significant balance problems compared to controls (PM group: median score 2, range 0–8 vs. control group: median score 0, range 0–3; *p* < 0.001) (Table 2).

## 4 Discussion

This study underscores the importance of monitoring FXAND-related symptoms in carriers of the *FMR1* PM. Although there is ongoing scientific debate concerning the clinical relevance of FXAND in this population, our findings suggest that structured anamnesis and targeted questioning are crucial for its accurate detection. In other words, these symptoms can be identified only if data are collected carefully and systematically during clinical visits. Otherwise, retrospective data collection under less controlled conditions often leads to a loss of essential information on FXAND manifestations, thereby significantly underestimating their true prevalence.

This study, which relied on prospective data collection through a structured interview, revealed that female PM carriers frequently experience FXAND-related symptoms, including chronic pain, chronic fatigue, anxiety, and depressive symptoms. Regarding chronic pain, shoulder girdle pain is more prevalent among female carriers. Moreover, our findings demonstrated a clear association between anxiety/depressive symptoms and chronic fatigue. Notably, although no official diagnoses of anxiety, depression, chronic pain syndrome, unspecified chronic fatigue, or fibromyalgia were recorded among study participants, PM carriers nonetheless reported symptoms consistent with each of these conditions. The very absence of formal diagnoses among participants should serve as a strong incentive for clinicians to collect clinical data more carefully, as a warning sign that insufficient attention is currently being paid to this spectrum of PM-related conditions (Klausner et al., 2025; Montanaro et al., 2025).

Our findings demonstrate that chronic pain is significantly more prevalent among PM carriers than in the control group, with over half of PM carriers reporting chronic pain compared to only quarter of controls. In addition to higher prevalence, PM carriers also reported significantly greater pain intensity, particularly for activities such as brushing or combing hair. Moreover, pain levels in the PM group tended to increase with age. These findings are consistent with previous research demonstrating increased pain sensitivity and higher rates of chronic pain conditions such as fibromyalgia in PM carriers (reviewed in Tassone et al., 2023). Coffey et al. (2008) identified increased rates of fibromyalgia in PM carriers with definite or probable FXTAS, while those without FXTAS exhibited higher rates of muscle pain, defined as injury-unrelated myalgia lasting longer than 2 months (Coffey et al., 2008). Similarly, Rodriguez-Revenga et al. (2009) reported that about 25% of female PM carriers over the age of 50 experienced chronic muscle pain, a significantly higher prevalence than the 2% observed in the general population of the same age group. However, the lack of a control group limited the broader conclusions of the study (Rodriguez-Revenga et al., 2009). In contrast, Hunter et al. (2010) did not find a difference in self-reported chronic pain between PM carriers and controls in the study involving 537 women (334 PM carriers) and 151 men (37 PM carriers), aged 18–50 years (Hunter et al., 2010). However, this study did not specifically assess pain or fibromyalgia in PM carriers, likely underestimating true prevalence. Notably, it did highlight that women with indicators of ovarian dysfunction reported higher rates of associated medical conditions (Hunter et al., 2010). Further support for the link between PM and chronic pain comes from a case series published by Leehey et al. (2011), which described five female PM carriers who exhibited fibromyalgia symptoms along with other central sensitivity syndromes (Leehey et al., 2011). In the majority of these patients, pain initially presented in the shoulders and arms and subsequently progressed to involve the entire body (Leehey et al., 2011). This pattern aligns with our observation that PM carriers more often reported discomfort during activities such as brushing or combing hair, suggesting shoulder girdle pain. Additionally, Johnson et al. (2022) conducted a study comparing males and females with FXTAS and found that female carriers experienced significantly more pain-related symptoms than males (Johnson et al., 2022). Women reported more frequent occurrences of allodynia, peripheral neuropathy pain, migraines, fibromyalgia, and back pain. They also exhibited significantly greater anxiety levels and used pain medication more frequently, further supporting the view that female PM carriers are particularly vulnerable to chronic pain syndromes (Johnson et al., 2022).

Moreover, both PM and intermediate *FMR1* allele carriers appear to exhibit connective tissue abnormalities and autonomic dysfunction, which may contribute to chronic and widespread pain beginning early in life (Butler et al., 2022; Tassanakijpanich et al., 2022). Although no previous research directly evaluated the association between pain severity and age in PM carriers, it is well established that aging in carriers who develop FXTAS, leads to progressive worsening of neurological and functional symptoms (Jacquemont et al., 2004). Consistent with previous research (Summers et al., 2014a), our findings revealed a significantly higher prevalence and severity of chronic fatigue among female PM carriers compared to controls. Specifically, over half of the PM carriers reported chronic fatigue, in contrast to only a tenth in the control group. Fatigue severity scores were also significantly elevated in PM carriers, both in overall scores and across specific dimensions of physical and mental fatigue. According to our research, PM carriers were more likely to report becoming tired easily, feeling physically exhausted, and lacking sufficient energy for daily life. Moreover, we observed no significant correlations between fatigue level and age, BMI, CGG repeat size, or the experience of raising a child with FXS. These findings align with those of Summers et al. (2014a), who reported greater fatigue severity in PM carriers with and without FXTAS compared to healthy controls (Summers et al., 2014a). Similarly, no significant difference in BMI was found between non-FXTAS carriers and controls, although PM carriers with FXTAS had a significantly higher average BMI compared to both non-FXTAS carriers and controls (Summers et al., 2014a).

The etiology of chronic fatigue in PM carriers remains multifactorial and incompletely understood. Mitochondrial dysfunction has been proposed as a key contributing mechanism: mitochondrial abnormalities, including impaired oxidative phosphorylation and elevated oxidative stress, are common in individuals with the PM and may underlie both fatigue and other systemic symptoms (Giulivi et al., 2016; Song et al., 2016). Furthermore, comorbidities such as sleep apnea, diabetes, and cardiovascular disease have been shown to exacerbate fatigue (Hamlin et al., 2011). Although diabetes and cardiovascular diseases were not specifically assessed in our sample, poorer sleep quality was more frequently reported in the PM group.

Anxiety symptoms were more prevalent in PM carriers than controls, but not significantly so. However, anxiety symptom severity was markedly higher among PM group, consistent with prior reports of elevated anxiety in this population, including generalized anxiety, social anxiety, and obsessive-compulsive traits, independently of FXTAS diagnosis (Cordeiro et al., 2015; Schneider et al., 2016; Tassone et al., 2023). Interestingly, our control group exhibited a relatively high prevalence of anxiety symptoms (62%), compared to the global prevalence estimate of around 7% (Baxter et al., 2013). In our study, anxiety symptom severity was not associated with CGG repeat length or having a child with FXS. This agrees with prior literature showing no relationship between anxiety and repeat length (Hunter et al., 2008) and demonstrating that anxiety can manifest in early childhood or adolescence, prior to motherhood (Cordeiro et al., 2015), as well as in PM women regardless of whether they have children affected by FXS (Gossett et al., 2016; Roberts et al., 2016). In contrast, one study showed that a higher risk of anxiety in the PM carriers was linked to having more children with FXS in the family (Roberts et al., 2009).

These findings diverge from the recent study of Klausner et al. (2025), who reported no association between PM and anxiety or ADHD in a cohort of over 53,000 women undergoing preconception carrier screening. Their analysis relied solely on electronic medical record (EMR) data, using diagnosis codes or prescription history to define neuropsychiatric phenotypes (Klausner et al., 2025). While the size of their dataset is a significant strength, their study design carries limitations. EMR-based methods may fail to capture subclinical, mild, or undiagnosed symptoms (Soto et al., 2024), particularly in psychiatric conditions that are often underreported or misdiagnosed in women, such as anxiety disorders and adult ADHD (Montanaro et al., 2025). In contrast, our study used direct participant interviews and validated self-report instruments, offering a more sensitive assessment of symptomatology (Spies et al., 2004). Notably, none of our participants had a formal diagnosis of anxiety, depression, or chronic pain, despite significantly elevated symptom severity scores. Many reported managing symptoms independently or not seeking professional care, supporting the likelihood that reliance on EMRs alone may underestimate the true burden of disease among PM carriers. Additional limitations of the study by Klausner et al. (2025), include treating “anxiety” as a single diagnosis without distinguishing between its subtypes, using antidepressant prescriptions as a proxy for anxiety, collapsing anxiety and depression into a single category, and grouping heterogeneous “other psychiatric disorders,” all of which may mask disorder-specific patterns (Montanaro et al., 2025). Klausner et al. also acknowledged that their sample may have been healthier than average, due to selection through a family-planning genetic screening program (Klausner et al., 2025).

In contrast to anxiety and fatigue, depressive symptoms did not differ significantly between PM carriers and controls; however, more than half of the PM and control groups reported experiencing depressive symptoms. Depressive symptoms severity did not correlate with age, CGG repeat size, BMI, or the child with FXS. While depressive symptom prevalence was similar across groups, when considering subclinical symptoms related to mood and motivation, such as mental exhaustion, reduced concentration, and decreased sense of accomplishment, PM carriers tended to report greater impairment than controls. However, these differences did not reach statistical significance. Our findings differ from several studies reporting that adult PM carriers, particularly women, are at increased risk for depression, a key component of the FXAND (Aishworiya et al., 2022; Tassone et al., 2023). Some studies have suggested a link between CGG repeat size and depression risk. For example, one study found a significantly higher risk of depression in PM carriers with over 100 CGGs (Johnston et al., 2001). At the same time, another observed a marginal association between repeat size and both depression and negative affect in males, and negative affect alone in females (Hunter et al., 2008). A further study in PM females identified a nonlinear relationship between CGG repeat size and depression, with the highest prevalence observed in those with mid-range repeats (85–110) (Seltzer et al., 2012b). Similarly, PM women with 70–100 repeats had the highest rates of DSM-defined major depressive disorder (MDD) (Roberts et al., 2009). Notably, many reported their first MDD episode prior to having a child with FXS, suggesting a biological predisposition rather than caregiving stress as the primary cause (Roberts et al., 2009). In contrast, other mood disorders such as dysthymia and bipolar disorder have not been reported at higher rates in PM carriers compared to controls (Bourgeois et al., 2009).

A key finding of the current study was the strong correlation between anxiety, depressive symptoms, and chronic fatigue in female PM carriers. In both the PM and control groups, anxiety and depressive symptom severity were positively correlated, reflecting the well-known comorbidity between these disorders (Kalin, 2020). Epidemiological data indicate that nearly half of individuals with lifetime MDD also had a history of one or more anxiety disorders (Kessler et al., 2015), with lifetime comorbidity ranging from 20 to 70% among various anxiety disorders (reviewed in Kalin, 2020).

In PM carriers specifically, anxiety frequently co-occurs with other health issues. Kenna et al. (2013) found comorbid anxiety and depression in 43% of PM mothers (Kenna et al., 2013). Furthermore, in our study, both anxiety and depressive symptoms were significantly associated with chronic fatigue in the PM group. Similarly, Kraan et al. reported high rates of social anxiety (approximately 38%) and depression (approximately 30%) linked to migraine and irritable bowel syndrome (Kraan et al., 2023). Previous research has also connected depression with increased fatigue in PM carriers, suggesting that effective treatment of depression may help reduce fatigue symptoms (Bourgeois et al., 2009; Summers et al., 2014b).

Interestingly, in our cohort, fatigue and chronic pain were not significantly associated in PM carriers, whereas a positive correlation between these symptoms was present in the control group. Furthermore, neither anxiety nor depressive symptoms were significantly correlated with chronic pain in PM carriers. In the general population, chronic pain and fatigue frequently co-occur, with central sensitization, impaired sleep, and inflammatory mechanisms serving as shared etiological factors (Louati and Berenbaum, 2015; Sluka and Clauw, 2016; Strand et al., 2020). A large meta-analysis encompassing over 347,000 adults with chronic pain across 50 countries found that approximately 39.3% exhibited clinically significant depressive symptoms, while 40.2% reported anxiety (Aaron et al., 2025). Supporting this, data from the 2019 U. S. National Health Interview Survey revealed that 23.9% of adults with chronic pain also experienced persistent symptoms of anxiety and/or depression, compared to only 4.9% of those without chronic pain (De La Rosa et al., 2024). Conversely, more than half of adults (55.6%) with anxiety and/or depression symptoms reported chronic pain (De La Rosa et al., 2024). These observations highlight the complex and often intertwined relationship among chronic pain, fatigue, anxiety, and depression, underscoring the importance of comprehensive assessment and integrated treatment approaches, particularly in vulnerable populations such as PM carriers.

Finally, this study revealed that the PM group exhibited significantly greater memory difficulties, frequent difficulty thinking clearly, and significant balance problems compared to controls. Because CNS changes in PM carriers gradually worsen over their lifetime compared to controls (Wang et al., 2017), it is possible that these neurological symptoms can represent prodromal symptoms of FXTAS or symptoms that may indicate a possible early development of FXTAS (Liani et al., 2025). Although no FXTAS patients were included in this study, the symptoms of memory problems, executive function deficits, and subtle balance problems in carriers can be associated with limited white matter disease on MRI, and these problems may represent prodromal features of FXTAS, whereas the full clinical features of tremor and ataxia and neuroradiological features are not met (Liani et al., 2025). Further longitudinal studies are needed to better understand who will progress to FXTAS and who will not.

In conclusion, this study provides evidence that female PM carriers experience a higher prevalence and severity of chronic pain (specifically shoulder girdle pain), chronic fatigue, and anxiety symptoms compared to age-matched non-carriers. In contrast, depressive symptoms are equally common in both groups. Chronic fatigue emerged as a significant clinical feature, strongly associated with both anxiety and depressive symptoms, whereas chronic pain was not significantly related to emotional distress in this cohort. These findings reinforce the concept of FXAND as a clinically relevant manifestation in a subset of female PM carriers, even in the absence of formal diagnoses or overt functional disability. The absence of documented diagnoses among participants highlights the need for greater awareness, proactive symptom screening, and a structured approach to anamnesis in this population. Specifically, systematic screening protocols for chronic fatigue, pain, and related emotional symptoms should be considered in clinical practice to facilitate early detection of FXAND. Early recognition of these features would enable timely, individualized interventions and support strategies, potentially prevent symptom escalation, and improve overall health outcomes. Finally, early identification and management of FXAND-related symptoms may help improve quality of life and reduce the risk of progression to more severe neuropsychiatric or somatic conditions.

A limitation of this study is the relatively small sample size, which may limit the generalizability of the results and reduce statistical power to detect subtle effects. An additional limitation is that multiple symptom domains were analyzed without formal correction for multiple comparisons, which, while intended to reduce the risk of Type II error in a small and correlated dataset, increases the risk of Type I error and warrants cautious interpretation of the findings. The reliance on symptom reporting during structured, in-person interviews, although conducted by a clinician-researcher with expertise in fragile X, may still be influenced by recall bias or imperfect memory, and the structured format might have shaped how participants interpreted and responded to certain questions, potentially affecting symptom reporting. Next, a limitation is that the interviewer was not blinded to participant group status, which may have introduced assessment bias. The control group demonstrated an unexpectedly high prevalence of anxiety symptoms, which could have diminished the observed differences between groups. The inclusion of relatives from families with fragile X as controls may introduce shared familial or environmental confounding factors, including subclinical neuropsychiatric traits; however, recruitment of unrelated control participants may be particularly challenging due to ethical considerations surrounding genetic testing in individuals who do not meet established clinical criteria for such testing. Future studies should employ longitudinal designs to determine whether specific symptom clusters, particularly those involving fatigue, predict the later development of neurological or psychiatric conditions in female PM carriers.

## Data Availability

The datasets presented in this study can be found in online repositories. The names of the repository/repositories and accession number(s) can be found in the article/Supplementary material.

## Footnotes

1
https://www.mapi-trust.org/
